# *PIK3CA* mutations are specifically localized to lymphatic endothelial cells of lymphatic malformations

**DOI:** 10.1371/journal.pone.0200343

**Published:** 2018-07-09

**Authors:** Hannah Blesinger, Silke Kaulfuß, Thiha Aung, Sonja Schwoch, Lukas Prantl, Jochen Rößler, Jörg Wilting, Jürgen Becker

**Affiliations:** 1 Institute of Anatomy and Cell Biology, University Medical School Göttingen, UMG, Göttingen, Germany; 2 Institute of Human Genetics, University Medical School Göttingen, UMG, Göttingen, Germany; 3 Center of Plastic, Hand and Reconstructive Surgery, University Medical Center Regensburg, Regensburg, Germany; 4 Clinics for Pediatric Hematology and Oncology, University Medical Hospital Freiburg, Freiburg, Germany; Peter MacCallum Cancer Centre, AUSTRALIA

## Abstract

Lymphatic malformations (LM) are characterized by the overgrowth of lymphatic vessels during pre- and postnatal development. Macrocystic, microcystic and combined forms of LM are known. The cysts are lined by lymphatic endothelial cells (LECs). Resection and sclerotherapy are the most common treatment methods. Recent studies performed on LM specimens in the United States of America have identified activating mutations in the phosphatidylinositol-4,5-bisphosphate 3-kinase catalytic subunit alpha (*PIK3CA*) gene in LM. However, whole tissue but not isolated cell types were studied. Here, we studied LM tissues resected at the University Hospitals Freiburg and Regensburg, Germany. We isolated LECs and fibroblasts separately, and sequenced the commonly affected exons 8, 10, and 21 of the *PIK3CA* gene. We confirm typical monoallelic mutations in 4 out of 6 LM-derived LEC lines, and describe two new mutations i.) in exon 10 (c.1636C>A; p.Gln546Lys), and ii.) a 3bp in-frame deletion of GAA (Glu109del). LM-derived fibroblasts did not possess such mutations, showing cell-type specificity of the gene defect. High activity of the PIK3CA—AKT- mTOR pathway was demonstrated by hyperphosphorylation of AKT-Ser473 in all LM-derived LECs (including the ones with newly identified mutations), as compared to normal LECs. Additionally, hyperphosphorylation of ERK was seen in all LM-derived LECs, except for the one with Glu109del. *In vitro*, the small molecule kinase inhibitors Buparlisib/BKM-120, Wortmannin, and Ly294002, (all inhibitors of PIK3CA), CAL-101 (inhibitor of PIK3CD), MK-2206 (AKT inhibitor), Sorafenib (multiple kinases inhibitor), and rapamycin (mTOR inhibitor) significantly blocked proliferation of LM-derived LECs in a concentration-dependent manner, but also blocked proliferation of normal LECs. However, MK-2206 appeared to be more specific for mutated LECs, except in case of Glu109 deletion. In sum, children that are, or will be, treated with kinase inhibitors must be monitored closely.

## Introduction

Lymphatic malformations (LM), also known as lymphangiomas, are characterized by the overgrowth of lymphatic vessels during pre- and postnatal development. They are most frequently found in the head and neck region but may be located in any organ containing lymphatics. Macro-cystic, micro-cystic and combined forms of LM are distinguished in the clinical diagnosis, the cysts being lined by lymphatic endothelial cells (LECs), which express typical LEC markers such as LYVE-1, Podoplanin (PDPN), PROX1, VEGFR-3 and CD31/PECAM-1 [1–5], although, however, the expression of PDPN/D2-40 has not been found in 4 of 12 LM [6]. The etiology of LM has long been under debate. Recent studies have identified activating mutations in the phosphatidylinositol-4,5-bisphosphate 3-kinase catalytic subunit alpha **(***PIK3CA)* gene in lymphovascular overgrowth disorders, with five specific mutations accounting for the majority of cases [7]. Such mutations, in combination with other oncogenic mutations, are also known to enhance tumor growth [8]. In a cohort of 31 LM patients from the Seattle Children’s Hospital, 74% showed activating *PIK3CA* mutations; and even more significantly, 16 out of 17 LM patients from the Boston Children's Hospital had *PIK3CA* mutations [7]. The tissues investigated in these studies contained many different cell types, and, although activating *PIK3CA* mutations have also been found in five LM-derived LEC lines isolated in the United States of America [9, 10], a direct comparison of different LM patient-derived cell lines has not been performed. We had access to tissue from 6 LM patients from the University Hospitals Freiburg and Regensburg, Germany. We isolated LM-derived LECs (Ly-LEC) and fibroblasts (Ly-F) and screened each cell type for the commonly affected exons 8, 10, and 21 of the *PIK3CA* gene. We identified 4 typical and two new activating mutations in the Ly-LEC lines, but never in fibroblasts, showing LEC-specificity of the mutation in LM. In search for specific inhibitors we treated Ly-LECs with 7 different kinase inhibitors, in comparison to normal foreskin-derived LECs. We observed significant reduction in proliferation of Ly-LECs with all of the inhibitors, but it must be pointed out that normal LECs behaved in the same or similar manner. Therefore, caution is advisable when treating young LM patients with kinase inhibitors, but a therapeutic window for such treatment may exist.

## Results

**Fig 1:**
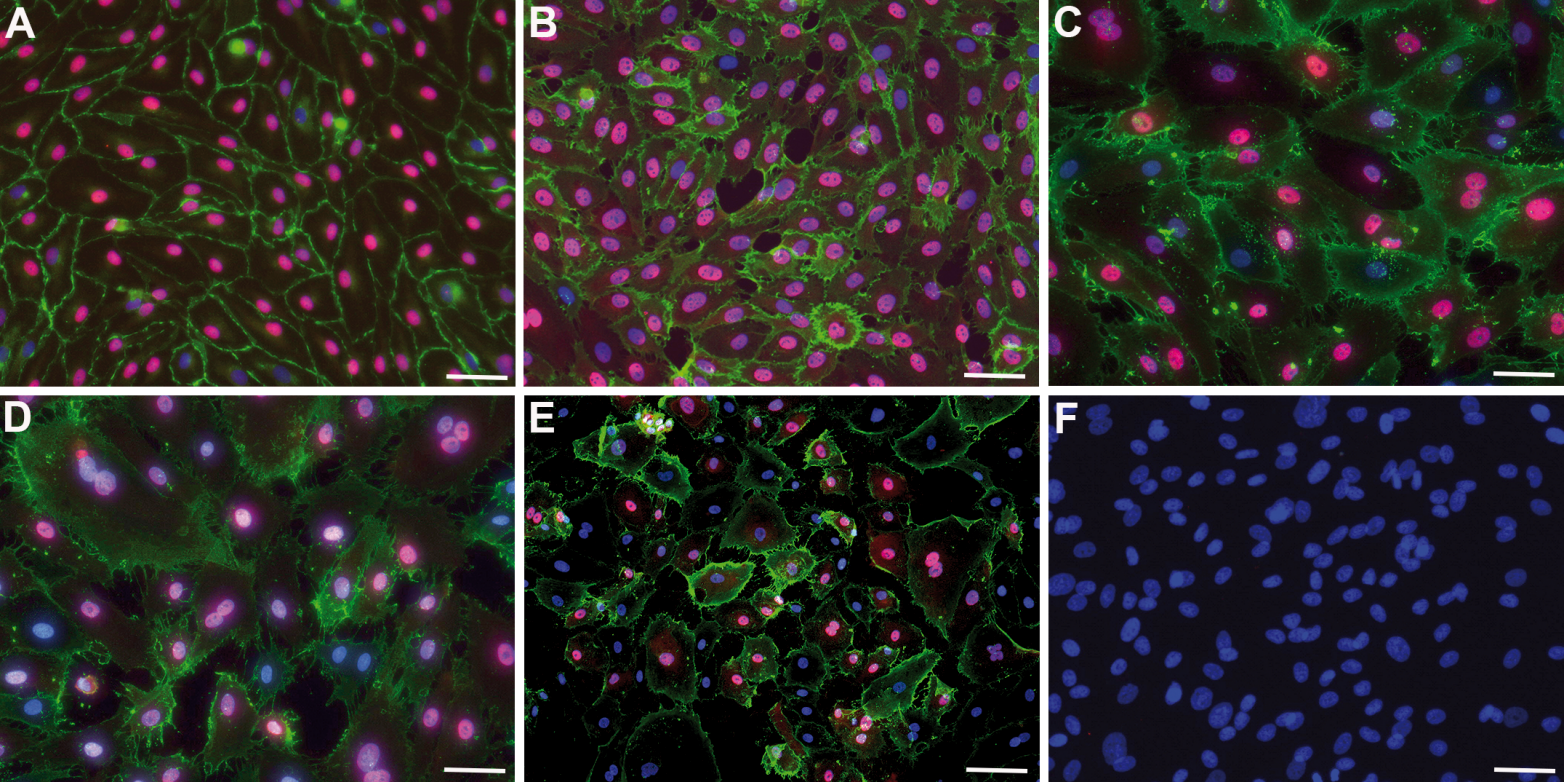
Culture of lymphatic endothelial cells and fibroblasts. Immunocytology showing CD31 (green) and PROX1 (magenta). Nuclei are counter-stained with DAPI (blue). **A**) Foreskin-derived HD-LECc4; **B**) Foreskin-derived HD-LECc2; **C**) Patient-derived Ly-LEC-12, **D**) Patient-derived Ly-LEC-2; **E**) Patient-derived Ly-LEC-10; **F**) Patient-derived fibroblasts (Ly-F-12) do not express CD31 or PROX1. Bar = 45 μm in A-D,F, and 90 μm in E.

**Fig 2:**
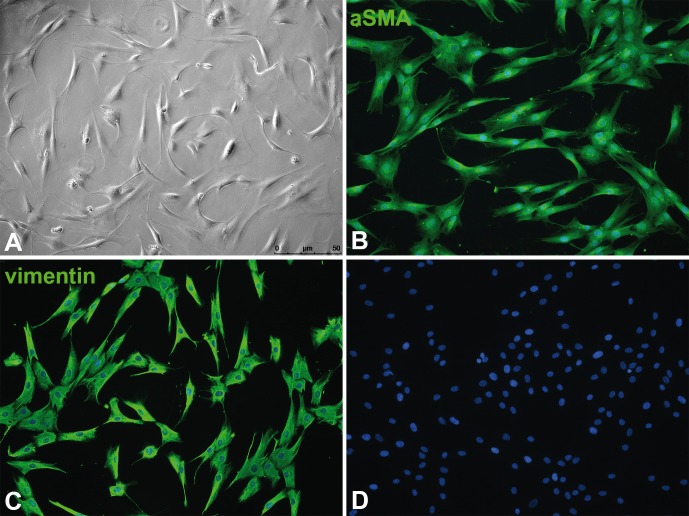
Patient-derived fibroblasts (Ly-F-12). **A**) Phase-contrast image showing typical morphology. **B**) Anti-αSMA staining. **C**) Anti-vimentin staining. **D**) Negative control without primary antibodies. Note that all fibroblasts express αSMA and vimentin. Bar = 50μm.

Recent studies have identified activating mutations in the *PIK3CA* gene in lymphovascular overgrowth disorders, with five specific mutations (in exons 8, 10, and 21) accounting for the majority of cases [7]. We isolated lymphangioma/lymphatic malformation (LM)-derived lymphatic endothelial cells (Ly-LEC) and fibroblasts (Ly-F) from 6 patients (Table 1). Growth characteristics and the expression of CD31 and PROX1 of Ly-LECs were compared to healthy human dermis/foreskin-derived HD-LECs. While HD-LECs showed a cobblestone morphology, CD31 expression in the cell membrane, and a robust nuclear PROX1 expression (Fig 1A and 1B), Ly-LECs showed a more variable PROX1 expression, heterogeneity in cell size, and sometimes a double nucleus (Fig 1C–1E). Patient-derived fibroblasts were characterized by the absence of CD31 and PROX1 (Fig 1F), typical morphology and growth characteristics, as well as their α-smooth muscle actin (αSMA) and vimentin expression (Fig 2).

**Table 1:** Mutation analysis of LM-derived cell-lines.

Cell line (alternative name)	Mutation (PIK3CA)	Gender	Age
Ly-LEC-1 (LEC-A; LEC-1)	Exon 8: c.1258T>C (p.C420R)	male	Infant
Ly-F-1	No mutation in exons 8, 10, 21		
Ly-LEC-2 (LEC-B; LEC-2)	3 bp in-frame GAA deletion; Glu109del **(*new*)**	male	Infant
Ly-F-2	No mutation in exons 8, 10, 21		
Ly-LEC-10	Exon 10: c.1636C>A (p.Q546K) **(*new*)**	female	Infant
Ly-F-10	No mutation in exons 8, 10, 21		
Ly-LEC-12	Exon 10: c.1633G>A (p.E545K)	female	Infant
Ly-F-12	No mutation in exons 8, 10, 21		
Ly-LEC-14	Exon 21: c.3140A>T, (p.H1047L)	female	Adult
Ly-F-14	No mutation in exons 8, 10, 21		
Ly-LEC-17	Exon 10: c.1633G>A (p.E545K)	male	Adult
Ly-F-17	No mutation in exons 8, 10, 21		
HD-LECc2	No mutation in exons 8, 10, 21	male	Infant
HD-LECc3	No mutation in exons 8, 10, 21	male	Infant
HD-LECc4	No mutation in exons 8, 10, 21	male	Infant

HD-LEC represent LECs from healthy donors. All other cells were isolated from LM patients. LY-LEC are LM-derived LECs, Ly-F are LM-derived fibroblasts.

We isolated genomic DNA and tested Ly-LECs and corresponding fibroblasts for activating *PIK3CA* mutations in exons 8, 10, and 21. In the first cell line (Ly-LEC-1), previously published as LEC-A or LEC-1 [11], we found the mutation c.1258T>C (p.C420R) in exon 8 (Table 1), which increases the enzyme’s baseline catalytic activity. We did not find mutations in fibroblasts (Ly-F-1) of the patient. In the second cell line, Ly-LEC-2, previously published as LEC-B/LEC-2 [11], we did not find a typical *PIK3CA* mutation in exons 8, 10, and 21, which also holds true for the fibroblasts from the same patient (Table 1). We therefore sequenced the whole *PIK3CA* gene in Ly-LEC-2 and found a 3bp in-frame GAA deletion in position 109 or 110 (there are two consecutive glutamic acids), previously described as Glu109del in carcinomas such as breast, endometrium, pancreas, and esophagus [12, 13]. Its effect on PIK3CA protein function, however, has remained unknown.

In Ly-LEC-10 we found a mutation in exon 10 (c.1636C>A; p.Gln546Lys), which has not been detected before in LECs, however, has been found in tumor cells [14, 15]. Again, its effect on PIK3CA protein function has remained unknown. In Ly-F-10 the mutation was not present. In Ly-LEC-12 and Ly-LEC-17 the mutation c.1633G>A (p.E545K) in exon 10 was found, which was not present in the fibroblasts from the same patients. In Ly-LEC-14 the mutation c.3140A>T, p.(H1047L) in exon 21 was found, and was not present in corresponding fibroblasts.

In sum, in 4 Ly-LEC lines we found an activating *PIK3CA* mutation. In two case (Ly-LEC2 and 10) we found a new mutation, with as yet unknown effects. Corresponding fibroblasts were always negative (Table 1).

**Fig 3:**
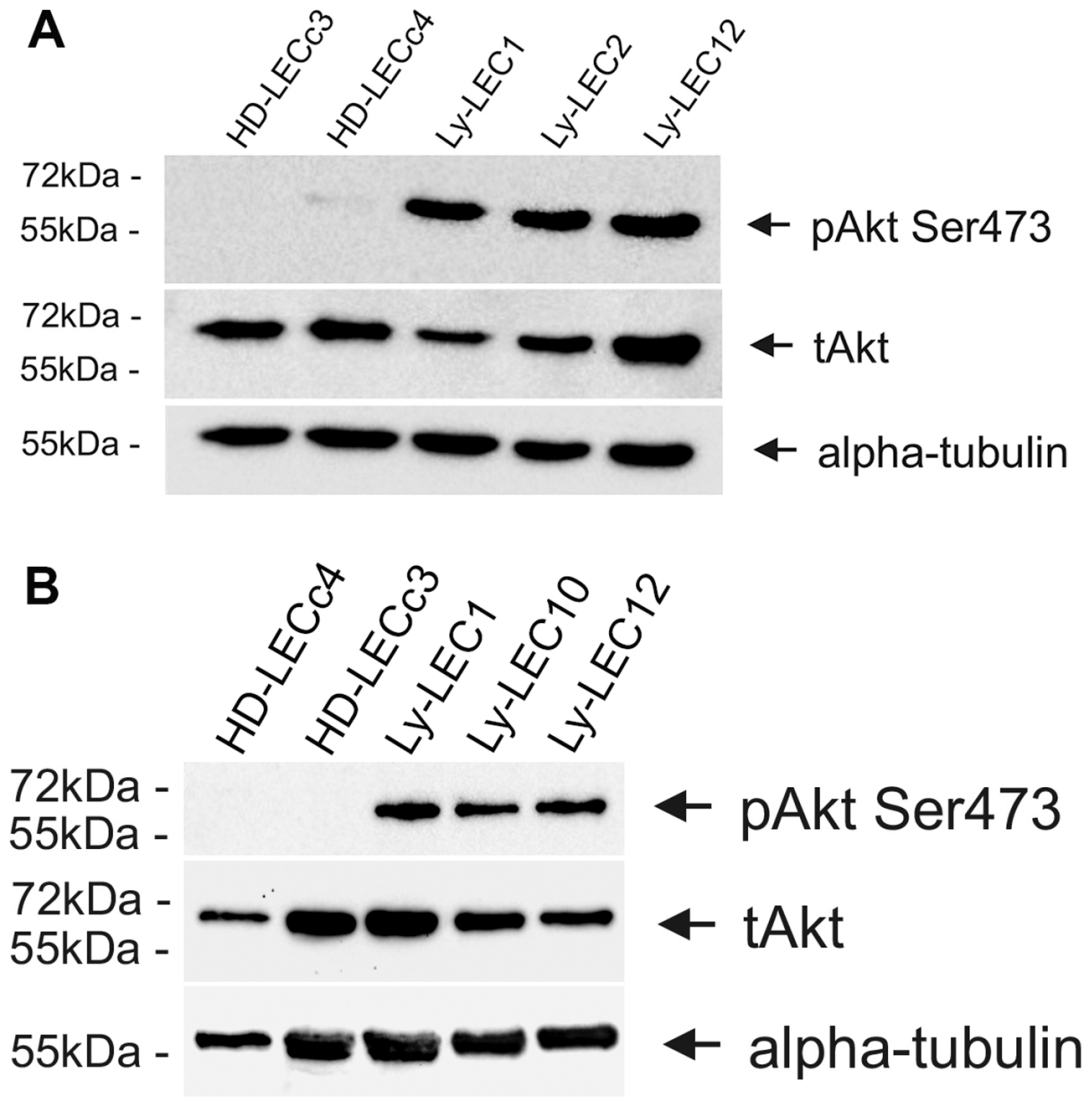
A,B. Phospho-AKT-Ser473 Western blot analysis of lymphatic endothelial cells. Note that phospho-AKT-Ser473 (pAKT) is highly expressed in lymphatic malformation-derived LECs (Ly-LEC1, 2, 10, and 12), but not in normal HD-LECc3 and c4. Total AKT (tAKT) is found in all cell lines. Antibodies against α-tubulin were used as loading control.

**Fig 4:**
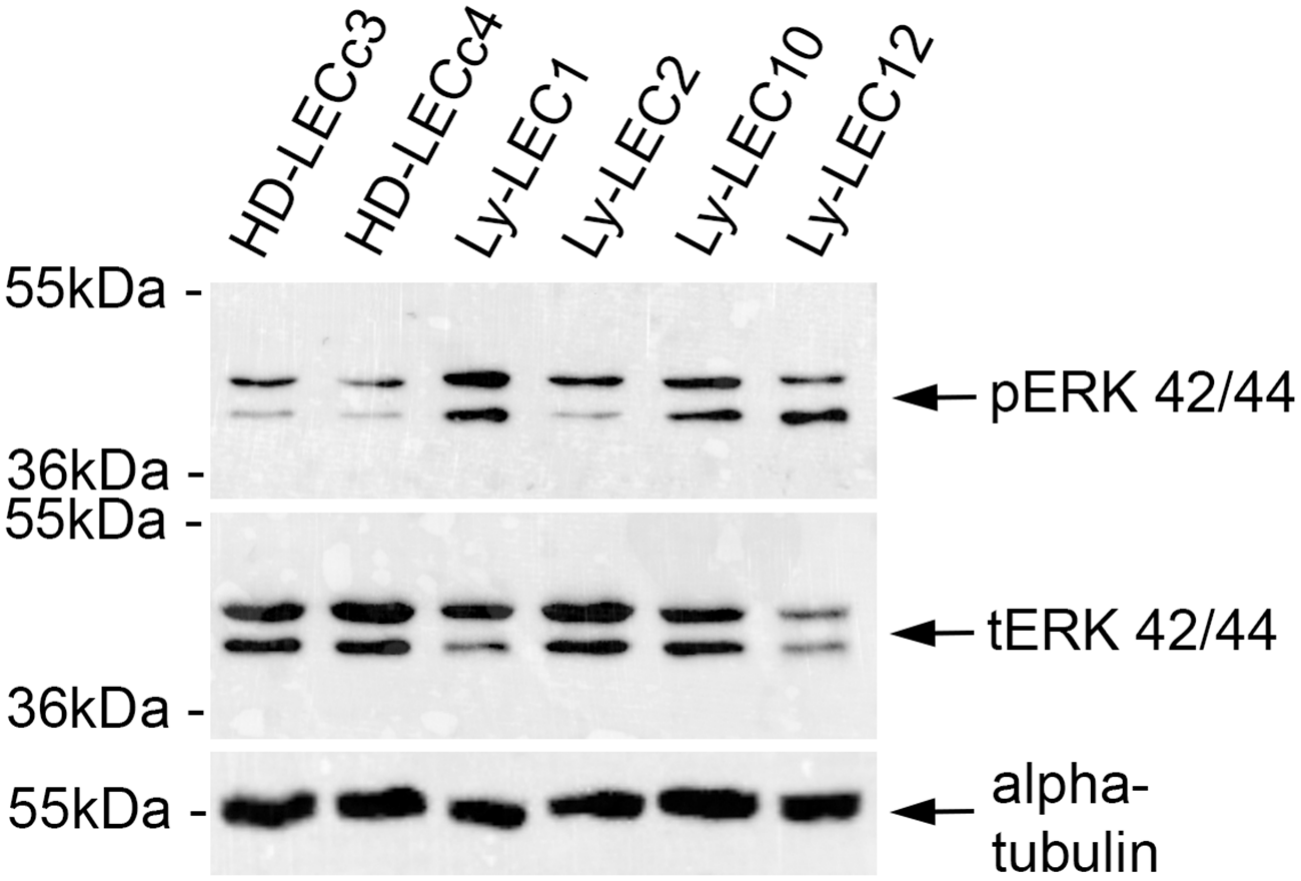
Phospho-ERK Western blot analysis of lymphatic endothelial cells. Note that phospho-ERK (pERK) is highly expressed in lymphatic malformation-derived Ly-LECs (Ly-LEC1, 10, and 12), but not in normal HD-LECc3 and c4. Ly-LEC2, which contain a Glu109del in *PIK3CA*, are similar to normal LECs, which contain clear levels of total ERK (tERK). Antibodies against α-tubulin were used as loading control.

Signal transduction of PIK3CA via the kinases AKT and mTOR has been well documented in tumor growth and angiogenesis [16]. We studied phosphorylation of AKT at position serine473 of Ly-LECs in comparison to normal HD-LECs and observed hyper-phosphorylation of AKT-Ser473 in all Ly-LECs, clearly showing their highly activated, angiogenic state (Fig 3). Thereby, AKT hyperphosphorylation was also seen in Ly-LEC2 and Ly-LEC10, which shows that the newly identified mutations also function as activating mutations. Phosphorylation of the mitogen-activated kinase ERK has also been shown to be a marker for angiogenic endothelial cells. We found hyperphosphorylation in all LM-derived LECs studied, except for Ly-LEC-2 (Fig 4), indicating a specific effect of Glu109del in LECs.

**Fig 5:**
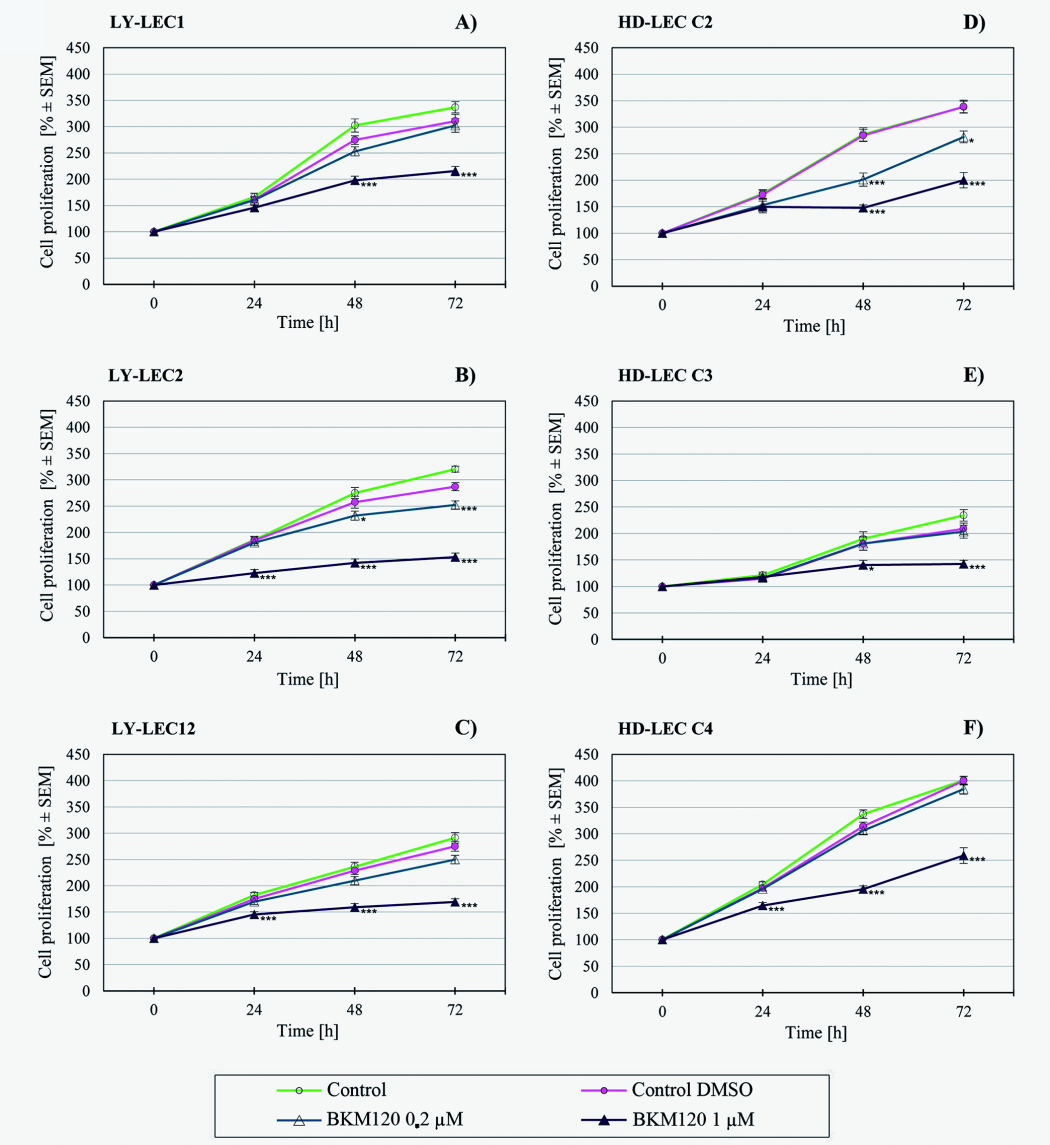
Proliferation studies with PIK3CA inhibitor Buparlisib (BKM120) on Ly-LEC (left side, A—C) and HD-LEC (right side, D—F), after 0, 24, 48 and 72 hours (h). 0 h values were set to 100%. Concentrations are indicated. Shown are the mean values of n = 3 independent experiments with each 5–8 replicates.

**Fig 6:**
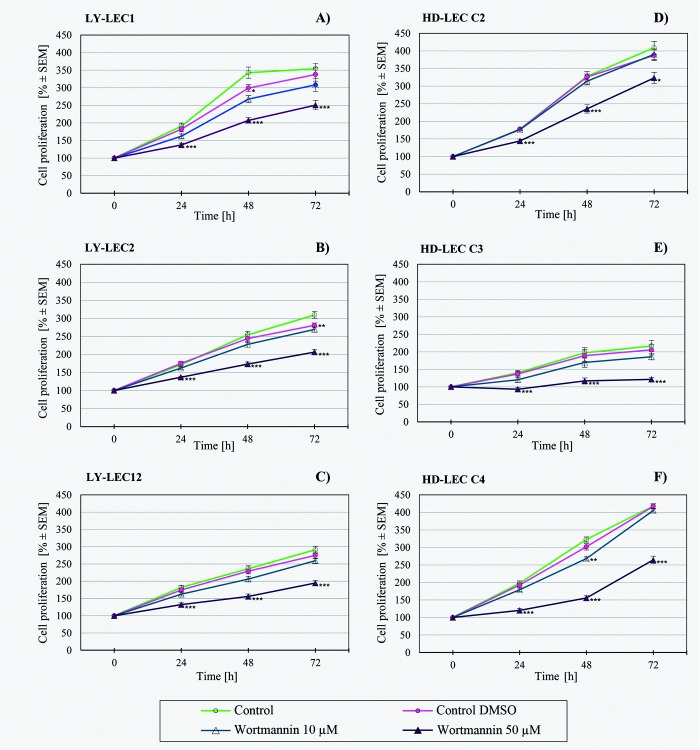
Proliferation studies with PIK3CA inhibitor Wortmannin on Ly-LEC (left side, A—C) and HD-LEC (right side, D—F), after 0, 24, 48 and 72 hours (h). 0 h values were set to 100%. Concentrations are indicated. Shown are the mean values of n = 3 independent experiments with each 5–8 replicates.

**Fig 7:**
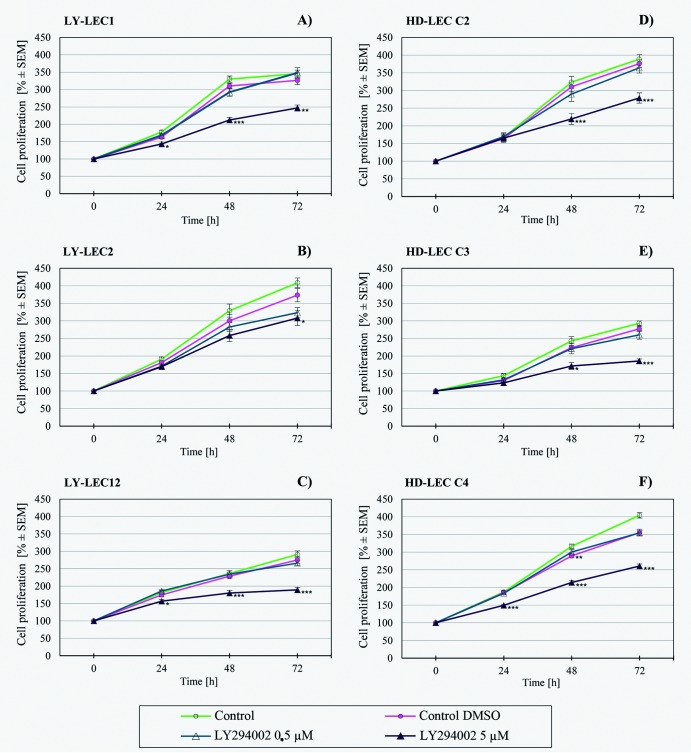
Proliferation studies with PIK3CA inhibitor Ly294002 on Ly-LEC (left side, A—C) and HD-LEC (right side, D—F), after 0, 24, 48 and 72 hours (h). 0 h values were set to 100%. Concentrations are indicated. Shown are the mean values of n = 3 independent experiments with each 5–8 replicates.

**Fig 8:**
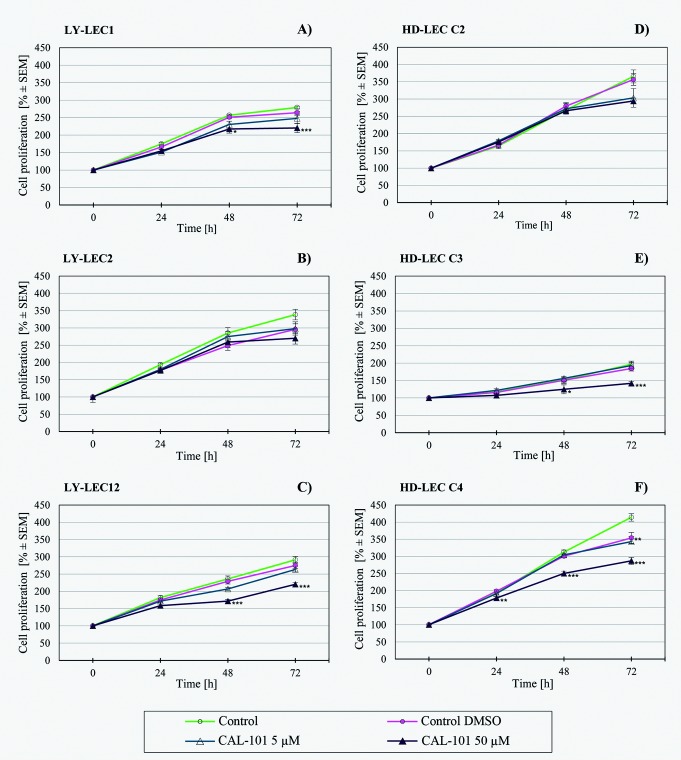
Proliferation studies with PIK3CD inhibitor CAL-101 (Idealisib) on Ly-LEC (left side, A—C) and HD-LEC (right side, D—F), after 0, 24, 48 and 72 hours (h). 0 h values were set to 100%. Concentrations are indicated. Shown are the mean values of n = 3 independent experiments with each 5–8 replicates.

**Fig 9:**
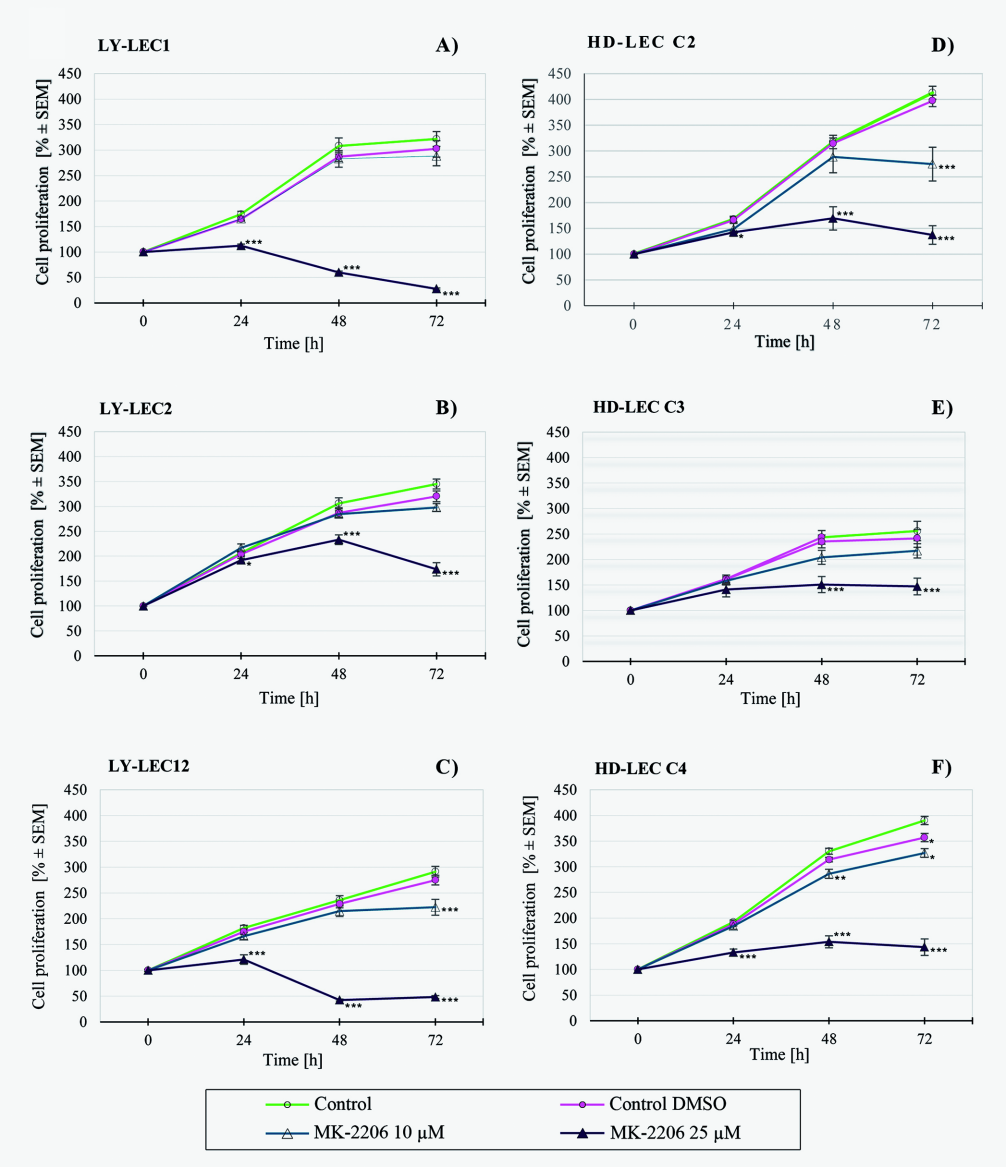
Proliferation studies with AKT inhibitor MK2206 on Ly-LEC (left side, A—C) and HD-LEC (right side, D—F), after 0, 24, 48 and 72 hours (h). 0 h values were set to 100%. Concentrations are indicated. Shown are the mean values of n = 3 independent experiments with each 5–8 replicates.

**Fig 10:**
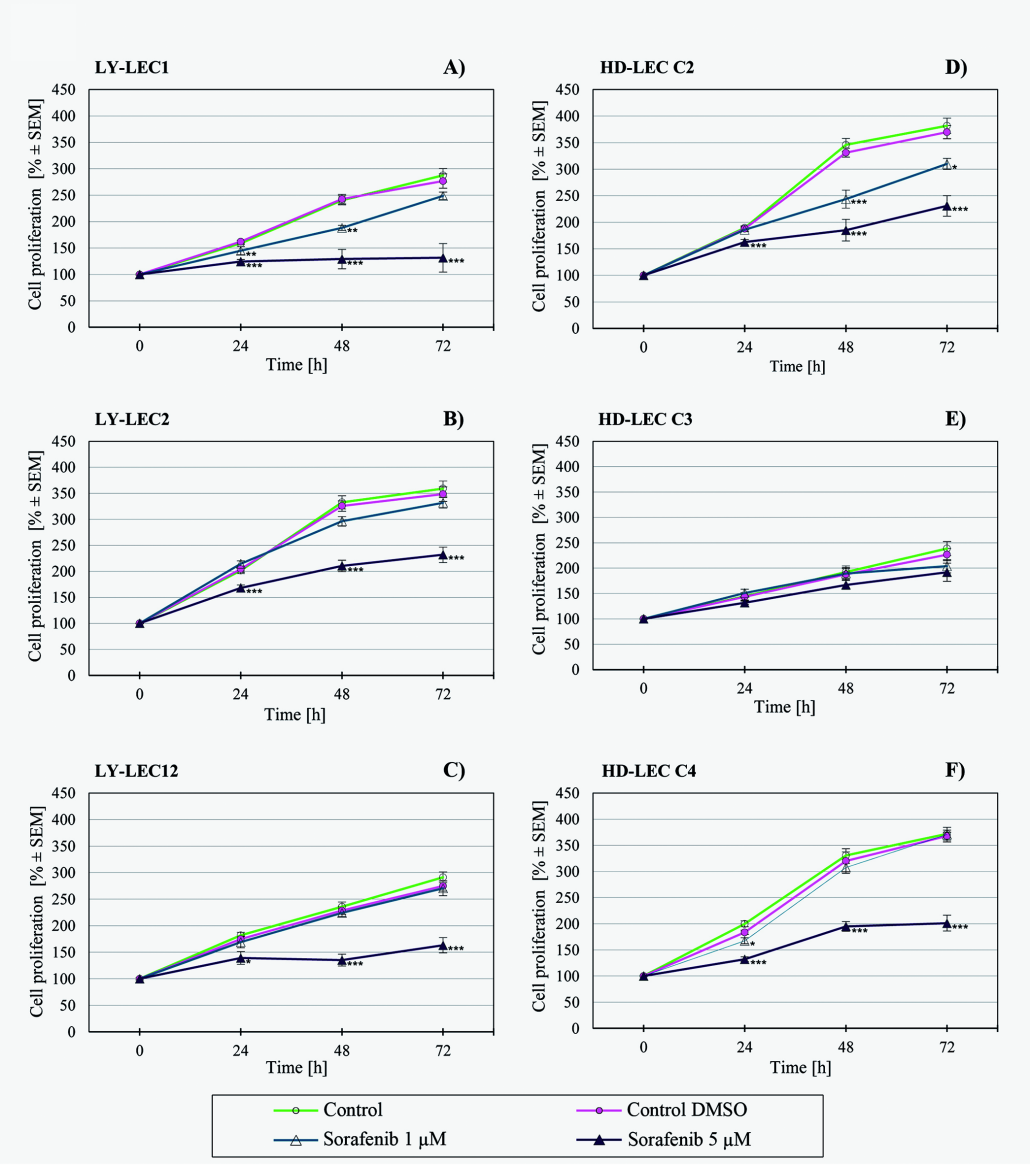
Proliferation studies with multi-kinase inhibitor Sorafenib on Ly-LEC (left side, A—C) and HD-LEC (right side, D—F), after 0, 24, 48 and 72 hours (h). 0 h values were set to 100%. Concentrations are indicated. Shown are the mean values of n = 3 independent experiments with each 5–8 replicates.

**Fig 11:**
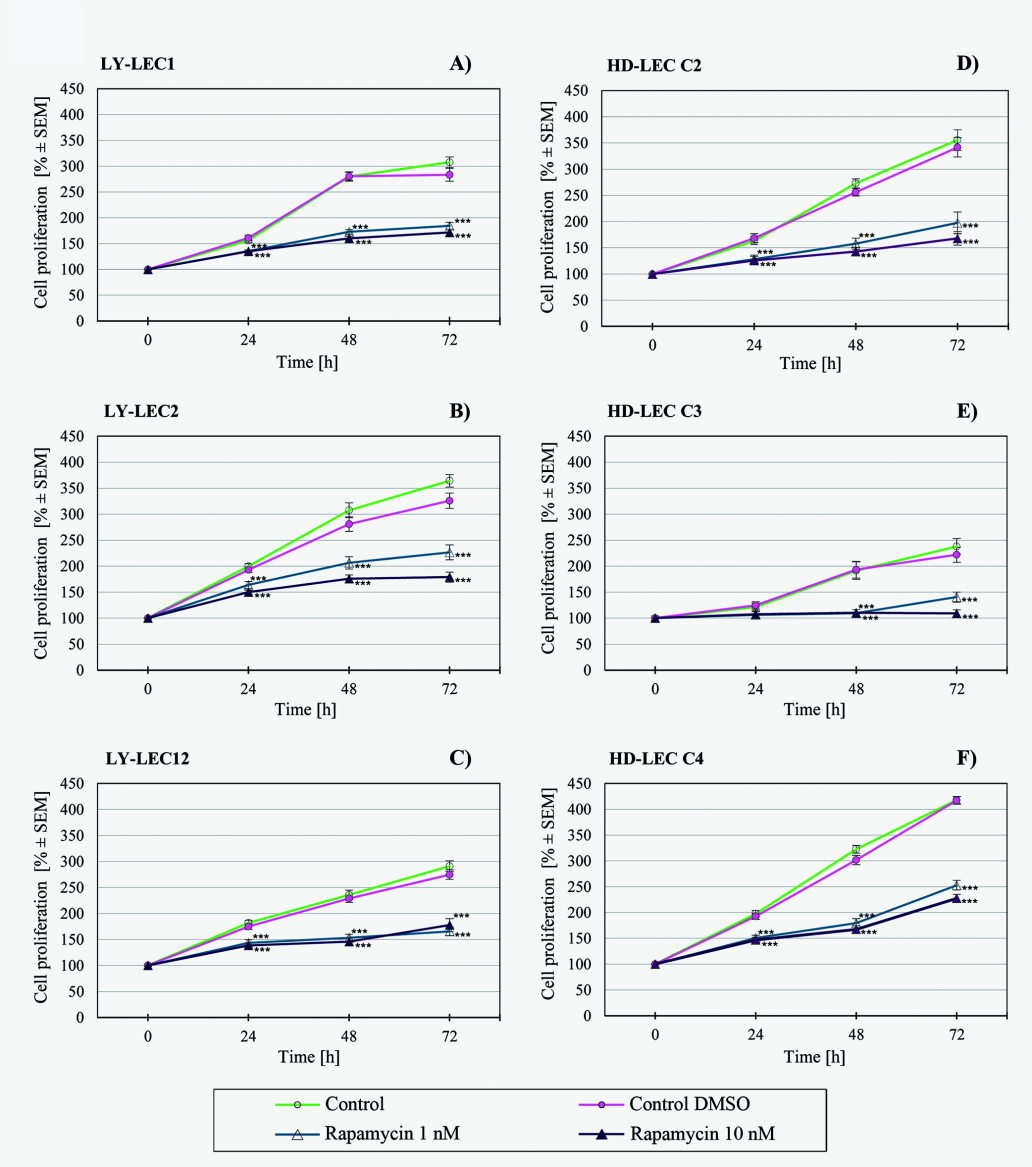
Proliferation studies with mTOR inhibitor rapamycin on Ly-LEC (left side, A—C) and HD-LEC (right side, D—F), after 0, 24, 48 and 72 hours (h). 0 h values were set to 100%. Concentrations are indicated. Shown are the mean values of n = 3 independent experiments with each 5–8 replicates.

In search for a kinase inhibitor with high specificity for *PIK3CA*-mutated LECs, we compared the anti-proliferative effects of 7 different kinase inhibitors on Ly-LECs (n = 3) and normal HD-LECs (n = 3). We studied Buparlisib (BKM120), Wortmannin, and Ly294002 (to inhibit PIK3CA), CAL-101/Idelalisib (to inhibit PIK3CD), MK-2206 (to inhibit AKT), Sorafenib (a multi-kinase inhibitor), and rapamycin (to inhibit mTOR). We observed significant dose-dependent growth inhibition with all inhibitors used, however, almost the same or similar responses were noted for normal HD-LEC (Figs 5–11). We did not immediately find an exclusive inhibitor for all LM-derived LECs, although it may be worth to test MK-2206 in greater detail. In 2 mutated cell lines (Ly-LEC1, Ly-LEC12) MK-2206 (25 μM) reduced cell numbers after 48h below the initial value. This was not the case in the 3 normal LEC lines (Fig 9). The only exception, Ly-LEC-2, seems to have an 'atypical' *PIK3CA* mutation, and shows no ERK hyperphosphorylation.

## Discussion

### Origin of lymphatic endothelial cells (LECs)

Vascular malformations (VM) can affect any type of vessel; blood vessels as well as lymphatics, isolated or in combined forms. A number of mutations, mostly loss of function mutations, have been identified to cause VM [17]. The combination of venous and lymphatic malformations is obviously rooted in the common origin of the two types of endothelial cells. Precursors for LECs reside in the lining of specific embryonic veins, but also in various mesenchymal compartments of vertebrate embryos [18, 4, 19]. Lymphangioblasts are still present in adult mouse lung [20], and also in the human, where they contribute to inflammation-induced lymphangiogenesis [21]. Smooth muscle cells in the *tunica media* of vessels originate from many different embryonic compartments [22]. In lymphatic collectors, they are in intimate contact with the luminal LECs, and, at transmission electron microscopic level, present as heterogenous population of cells [23]. Among the indispensable regulators of lymphangiogenesis are Vascular Endothelial Growth Factor-C (VEGF-C) and its high affinity receptor VEGFR-3 [24, 25, 4]. Loss of function mutations of VEGFR-3 are the main cause of congenital (primary) lymphedema, due to hypoplasia of the lymphatics [26].

### Activating PIK3CA mutations in LECs of lymphatic malformations

The reasons for hyperplasia of lymphatics have long been unknown, however, recently it has been shown that lymphatic malformations (LM), isolated and in combination with other overgrowth syndromes, are mostly caused by activating mutations in the *PIK3CA* gene. Mutations have been found in 74% of 31 individuals with isolated LM in a cohort from the Seattle Children’s Hospital [7], and in a cohort from the Boston Children’s Hospital even >90% (16 of 17) LM were somatic mosaic for specific *PIK3CA* mutations. However, no significant correlation between type of mutation, macro-cystic or micro-cystic LM, or clinical severity could be observed [7]. Similarly, when LM is part of a complex syndrome, *PIK3CA* is of major importance. It has been shown that 19 of 21 individuals with Klippel-Trenaunay syndrome (KTS), 5 of 8 with fibro-adipose vascular anomaly (FAVA), and 31 of 33 with congenital lipomatous overgrowth with vascular, epidermal and skeletal anomalies (CLOVES) were mosaic for specific *PIK3CA* mutations [7]. Thereby, 5 recurrent mutations have been found in the majority (~80%) of cases, resulting in amino acid exchange p.C420R, p.E542K, p.E545K, p.H1047R, and p.H1047L [7]. However, the authors also noted that the number of mutated cells in the LM tissue was less than 10%.

Therefore, we isolated both LECs and fibroblasts from LM tissues of 6 patients, and screened the commonly affected exons 8, 10, and 21 of the *PIK3CA* gene. Of note, a change in exon numbering of *PIK3CA* has taken place. Since exon 1 is entirely UTR, numerous previous studies have numbered the exons according to the coding exons. As a result, these studies refer to E545 mutations as being in exon 9 and H1047 mutations in exon 20 [27]. However, according to the recommendations of the Human Genome Variation Society (HGVS) exon numbering should always start with exon 1 as it appears, including also non-coding exons (www.hgvs.org/mutnomen/refseq.html#exinnr).

For separation of LM-derived LECs and fibroblasts we performed a 'classical' mechanical isolation procedure, the cell sweeping procedure, originally described by Dr. Judah Folkman [28]. The reason for not performing immuno-isolation techniques with surface molecules such as Podoplanin (PDPN) and LYVE-1 is an obvious one. LM are often derived from lymphatic collectors. Importantly, collector LECs, but also numerous lymph node LECs [29] are negative for the two markers [2, 23], which also applies to 1/3 of LM [6]. The most reliable marker for LECs from all parts of the lymphovascular system, including the lymph nodes, is double-staining for CD31 and Prox1 [30, 29, 23]. This, together with Dapi, enables counting of positive vs. negative cells, and exact quantification. Remaining cells are mostly fibroblasts, which escaped the cell sweeping. Previous studies who have used CD31 plus PDPN sorting of LM-LECs [9, 10] have obviously selected for lymphatic capillary-derived LECs and missed collector-derived LECs.

We identified 1 new and 4 typical activating mutations in these exons, as well as a new in-frame Glu109 deletion in Ly-LECs, but never in fibroblasts. This clearly shows that the mutations are cell-type-specific. In two Ly-LEC lines we found new mutations. Thereby, the cell line Ly-LEC2 showed a Glu109 deletion, which results in the loss of an amino acid in the region linking PI3K-ABD and PI3K-RBD domains (https://ckb.jax.org/geneVariant/show?geneVariantId=3785). Such deletion has been found, among other multiple mutations, in several carcinomas [12, 13], but its effect on protein function has remained unknown. Here, we have shown AKT-Ser473 hyperphosphorylation, but absence of ERK hyperphosphorylation, of Ly-LEC2, indicating that Glu109del serves as an activating mutation, which however differs in its signaling cascade from the other activating mutations. In Ly-LEC-10 we found a mutation in exon 10 (c.1636C>A; p.Gln546Lys = p.Q546K), which has also not been detected in Ly-LECs before. Again, this mutation has previously been found in tumors such as ovarian tumors, glioblastoma, uterine carcinosarcoma, and squamous cell carcinoma [14, 15]; also see: https://www.ncbi.nlm.nih.gov/pubmed/26619011). Both AKT-Ser473 and ERK hyperphosphorylation in Ly-LEC10 shows that this mutation represents an activating mutation as well.

Previously, the p.H1047L mutation in the *PIK3CA* gene, in addition to a premature stop codon in the PI3K regulatory subunit-3 (*PIK3R3*), has been found in a LM-derived LEC line isolated at the Boston Children’s Hospital [9]. The authors also observed high angiogenic potential of this cell line *in vitro*, as well as hyperphosphorylation of AKT-Thr308. Correspondingly, in another four LM-derived LEC lines isolated at the Cincinnati Children’s Hospital, p.E542K and p.E545A mutations, lymphangiogenic phenotype and AKT activation were identified [10]. We found hyperphosphorylation of AKT-Ser473 in LM-derived LECs, which supports the well-known observation of the activation of angiogenesis via the PI3K—AKT—mTOR pathway [16]. Activating mutations in the *PIK3CA* gene have very recently also been found in patients with venous malformations [31, 32], underlining the need for a causal therapy of vascular malformations, e.g. with kinase inhibitors.

We have therefore tested a panel of 7 kinase inhibitors on LM-derived Ly-LEC in comparison to normal HD-LECs. We studied BKM-120, Wortmannin, Ly294002, which are inhibitors of PIK3CA, CAL-101 (Idelalisib), an inhibitor of PIK3CD, MK-2206, an AKT inhibitor, Sorafenib, a multi-kinase inhibitor, and rapamycin, which inhibits mTOR. We observed significant dose-dependent growth inhibition with all inhibitors used, however, normal LECs responded similarly. Of note, the PIK3CD inhibitor CAL-101 [33] showed no effects, or effects only at very high dosage, which underlines the fact that PIC3CA, but not PIC3CD, is involved in the disease.

In contrast to a previous study, which reported on specific rapamycin effects on LM-derived LECs (a single cell line was used), but not healthy LECs [9], we did not detect an immediately obvious therapeutic window *in vitro*, neither for rapamycin, nor for the other kinase inhibitors tested, with the exception that, however, MK-2206 might be worth to be further tested with 'typical' lymphatic malformations. After 48h, we observed reduction of cell numbers below the initial value in 2 out of 3 LM-derived Ly-LEC lines with 25μM MK-2206, but not in healthy HD-LECs. Thereby, the LM-derived LEC line that did not respond so well was Ly-LEC-2, which possesses the Glu109 deletion and no ERK hyperphosphorylation. Tests with other Ly-LEC lines will have to determine if MK-2206 might be the inhibitor of choice for patients with *PIK3CA* mutations accounting for the majority of cases. Thereby, the possibility that a second mutation may accompany the *PIK3CA* mutation has to be taken into account [34].

Can the *in vitro* data be directly transferred to the situation in patients, to infants or adults? It is known that blood vascular endothelial cells in adults hardly proliferate [35]. To the best of our knowledge, proliferation of blood vascular and lymphatic endothelial cells has never been studied in children, but there seems to be no doubt that the values are significantly higher. With immunofluorescence we have previously observed expression of VEGFR-2 and -3 in blood vessels and lymphatics of infants [2], which underlines the immaturity of vessels in early years after birth. Nevertheless, the turnover of endothelial cells in infants is likely to be significantly lower than the proliferation of the cells *in vitro*. Therefore, our *in vitro* data may not be directly transferrable to the *in vivo* situation, and a therapeutic window for kinase inhibitors may well be present. It goes without saying that children must be closely monitored under treatment. Even more likely, a therapeutic window may be present for the treatment of adult LM-patients.

In summary, our studies show that activating *PIK3CA* mutations in LM are specifically localized in LECs. We identified a new mutation in exon 10 and a Glu109 deletion of the gene and show that several kinase inhibitors have the potential to block LEC proliferation. However, since these effects are not restricted to mutated cells, caution is advisable when treating young patients.

## Materials and methods

### Isolation and culture of cells

The Ethics Committee of the University Medical Hospital Göttingen approved this study (application no.: 18/1/18). Studies were performed with the informed and written consent of the patients or their legal representatives. Lymphangioma/LM-derived LECs (Ly-LECs) and LM-derived fibroblasts (Ly-F) were isolated as described [11]. Briefly, we used the cell sweeping procedure, a purely mechanical method which was originally introduced by Judah Folkman [28]. LM tissue was cut into small pieces and cultured in endothelial growth medium (MV2), including supplements, fetal bovine serum (PromoCell, Heidelberg, Germany), and 1% penicillin/streptomycin (Thermo-Fisher, Darmstadt, Germany). Ly-LECs and fibroblasts (Ly-F) that migrated out were separated mechanically, and cells were further cultured at 37°C and 5% CO_2_. The lymphendothelial identity was regularly controlled by immunocytology with antibodies against CD31/PECAM-1 (BD, Heidelberg, Germany) and PROX1 (ReliaTech, Wolfenbüttel, Germany). Purity of the cells was at least 80–90%. Pure LM-derived fibroblasts (Ly-F) were cultured in RPMI 1640 (Lonza, Basel, Switzerland), and characterized by their morphology, growth behavior and antibodies against vimentin and α smooth muscle actin (αSMA). Normal foreskin-derived LECs (HD-LEC) were bought from PromoCell (Heidelberg, Germany) and controlled by their CD31/PROX1 expression. Cells were cultured as described above.

### Mutation analysis of Ly-LEC and Ly-F

Genomic DNA of LM-derived cell lines (Ly-LEC, Ly-F) and normal lymphatic endothelial cells (HD-LEC) was extracted using DNeasy blood & tissue kit (Qiagen) according to the manufacturer’s protocol. For sequence analysis, exons 8, 10 and 21 (≙ coding exons 7, 9 and 20) of *PIK3CA* (phosphatidylinositol-4,5-bisphosphate 3-kinase, catalytic subunit alpha; NM_006218.2) were amplified from the genomic DNA using the primer sets depicted in Table 2. For exon 10, it was verified not to amplify parts of the pseudogene. PCR products were purified and used in the sequencing reaction, which included BigDye and sequencing primers, and was performed on ABI 3500 (Applied Biosystems, Thermo-Fisher, Foster City, CA). Data were analyzed using the software SeqPilot (JSI medical systems GmbH, Ettenheim, Germany).

**Table 2:** Primers used for mutation analyses of *PIK3CA*.

Primer	Fw (5’– 3’)	Rev (5’– 3’)
Exon 8	GGTCAGCTCTCCCAGCGATGGAAAGAATG GGCTTAAACCTTG	CAGCTCTCCCACAGGCGAATAAGAGAG AAGGTTTGACTGCC
	GGTCAGCTCTCCCAGCGATAGGAAAGAAT GGGCTTAAACCT	CAGCTCTCCCACAGGCGAAAAGAGAG AAGGTTTGACTGCCA
Exon 10	GGTCAGCTCTCCCAGCGATAGATTGGTTC TTTCCTGTCTCTGA	CAGCTCTCCCACAGGCGAATGAGATCA GCCAAATTCAGTTATTT
		CAGCTCTCCCACAGGCGAACATTTAAT GTGCCAACTACCAATGT
Exon 21	GGTCAGCTCTCCCAGCGATCCTGAAGGTA TTAACATCATTTGCTCC	CAGCTCTCCCACAGGCGAAGCCTGCTG AGAGTTATTAACAGTGC
Seq_R	CAGCTCTCCCACAGGCGAA	
Seq_F	GGTCAGCTCTCCCAGCGAT	

### Immunocytology

Cells were seeded on chamber slides (BD). After 24 hours, immunostaining was performed as follows: after short (1 min) 4% paraformaldehyde (PFA) fixation, cells were washed with PBS and incubated in bovine serum albumin (BSA). Then 0.1% Tween/PBS (for better PROX1 staining results—30 seconds) was applied, followed by immunostaining. Anti-human PROX1 (1:200, ReliaTech, Wolfenbüttel, Germany), anti-human CD31 (1:50; BD, NJ, USA), mouse-anti-human vimentin (1:1, DAKO; clone V9), and mouse-anti-human smooth muscle α-actin (αSMA) (1:500, Sigma, clone 1A4) antibodies were incubated for one hour, rinsed with PBS, and then secondary antibody staining with Alexa594-conjugated goat-anti-rabbit and Alexa488-conjugated goat-anti-mouse (1:200; ThermoFisher Scientific, MA, USA) was performed. Cells were counter-stained with Dapi and analyzed with AxioImager Z.1 (Zeiss, Göttingen, Germany).

### Western blot

Cells were washed twice with 5 ml DPBS (Pan Biotech; Aidenbach, Germany) and 1 mM sodium orthovanadate (Sigma-Aldrich; Steinheim, Germany) and subsequently lysed with RIPA buffer for 10 minutes on crushed ice. Samples were transferred to micro tubes (Sarstedt; Nürnberg, Germany) and centrifuged by 20,800x g for 15 minutes. Protein lysates were collected and used for SDS-PAGE according to standard procedures (Bio-Rad Laboratories; München, Germany). Proteins were transferred to PVDF membranes (Roth; Karlsruhe, Germany), which were then washed with TBST (0.1% Tween 20) for 10 minutes and blocked for one hour in blocking buffer (5% BSA in TBST 0.1%). Primary antibodies were: pAKT-Ser473, 1:500 in 5% BSA (rabbit mAb: Cell Signaling Technology, #4060); total AKT (rabbit pAb: Cell Signaling, #9272, 1:1000); phospho-p44/42MAPK (pERK1/2), Thr202/Tyr204, (rabbit mAb: Cell Signaling, #4370); p44/42MAPK (ERK1/2) (rabbit mAb: Cell Signaling, #4695); and peroxidase-conjugated α-tubulin, 1:10000 (Abcam, ab40742). Incubation was at 4 °C overnight. After washing with TBST (0.1% Tween 20) for 10 min., the peroxidase-conjugated secondary anti-rabbit IgG, 1:2000 (Santa Cruz; USA) were incubated for one hours. After washing, antigen-antibody complexes were visualized with ECL and Chemidoc Touch Imaging System (Bio-Rad).

### Proliferation assays

Proliferation assays were performed as described [36]. Briefly, 5000 cells were seeded into 96-well cell culture plates (Sarsted, Nümbrecht, Germany), and allowed to adhere overnight. Inhibitors or solvent controls were added at indicated concentrations. Cells were fixed with 4% glutaraldehyde after 24, 48 and 72 hours, stained with crystal violet (0.01% in deionized water), and absorption was measured at 570 nm with a microplate reader. Data are presented as the mean values of n = 3 independent experiments with each 5–8 replicates.

### Kinase inhibitors

All kinase inhibitors were bought from Seleckchem (Absource Diagnostics, Munich, Germany). We used Buparlisib/BKM120, Wortmannin, and Ly294002 (to inhibit PIK3CA), CAL-101/Idelalisib (to inhibit PIK3CD), MK2206 (AKT inhibitor), Sorafenib (multi-kinase inhibitor), and rapamycin (mTOR inhibitor). Inhibitors were diluted in DMSO (Sigma-Aldrich, Taufkirchen, Germany). Controls were performed with DMSO, corresponding to the highest concentration used in experimental groups.

### Statistical methods

Statistical analyzes were conducted by two-way-ANOVA with STATISTICA software (Statsoft, Tulsa, Oklahoma). Normal distribution was verified before testing. The standard error of the mean (SEM) was calculated and is shown in the graphs. Significant differences were considered as p-value ≤ 0.05 [*], p ≤ 0.01 [**] and p ≤ 0.001 [***].

## Abbreviations

AKT: AKT subfamily of serine/threonine kinases
CD31/PECAM-1: Platelet endothelial adhesion molecule-1
CLOVES: Congenital lipomatous overgrowth with vascular, epidermal and skeletal anomalies
EGM-2: Endothelial growth medium-2
ERK: Extracellular signal-regulated kinases
FAVA: Fibro-adipose vascular anomaly
HD-LEC: Human dermal lymphatic endothelial cell
KTS: Klippel-Trenaunay syndrome
LEC: Lymphatic endothelial cell
Ly-F: Lymphatic malformation (lymphangioma)-derived fibroblast
Ly-LEC: Lymphatic malformation (lymphangioma)-derived lymphatic endothelial cell
LYVE-1: Lymphatic vessel endothelial hyaluronic acid receptor-1
mTOR: Mechanistic target of rapamycin
PDPN: Podoplanin
PIK3CA: Phosphatidylinositol-4,5-bisphosphate 3-kinase, catalytic subunit alpha
PROX-1: Prospero-related homeobox transcription factor-1
VEGF-C: Vascular endothelial growth factor-C
VEGFR-3: Vascular endothelial growth factor receptor-3
